# Hydrodissection to create conjunctival flaps in dogs with corneal ulcers

**DOI:** 10.14202/vetworld.2023.2457-2463

**Published:** 2023-12-20

**Authors:** Lygia Silva Galeno, Alice Regina Silva Lopes, José Ribamar da Silva Júnior, Ana Lúcia Abreu-Silva, Tiago Barbalho Lima

**Affiliations:** 1 Graduated Program of Animal Science, State University of Maranhão, São Luís, Brazil; 2 Veterinary Teaching Hospital, State University of Maranhão, São Luís, Brazil; 3 Departament of Veterinary Medicine, State University of Maranhão, São Luís, Brazil; 4 Departament of Pathology, State University of Maranhão, São Luís, Brazil.

**Keywords:** conjunctival hydrodissection, corneal ulcer, divulsion

## Abstract

**Background and Aim:**

Hydrodissection is a liquid injection technique that is rarely used in animal ophthalmic procedures. The use of this technique in the creation of conjunctival flaps for the treatment of corneal ulcers in dogs can improve the outcome, task, and comfort for patients. This study aimed to evaluate the use of hydrodissection in the creation of conjunctival flaps in dogs with corneal ulcers.

**Materials and Methods:**

This study focused on a surgical procedure for creating conjunctival flaps in the eyes of 17 dogs with deep corneal ulcers. We divided the patients into two groups: Hydrodissection was performed in the first group (G1) and conventional divulsion without hydrodissection in the second group (G2). In G1, the conjunctival flap was created by subconjunctival injection of 1 mL of 0.9% sodium chloride, followed by flap construction. The flap was constructed through conventional divulsion using iris scissors in the G2. The operative time, degree of bleeding, and ease of conjunctival divulsion were evaluated during the procedure. Blepharospasm, hyperemia, edema, and scarring of the conjunctiva were evaluated during the post-operative period. Post-operative complications, notably suture dehiscence, were recorded in each group.

**Results:**

Hydrodissection is an easy-to-perform maneuver that optimizes the construction of conjunctival flaps. There were no statistical differences in the parameters used to evaluate the trans- and post-operative period between the groups. The volume of sodium chloride administered in the conjunctiva ranged from 0.5 mL to 1 mL in G1. Dehiscence of the flap sutures was observed in four patients (two in G1 and two in G2), with no significant difference between the groups.

**Conclusion:**

Hydrodissection facilitates the construction of conjunctival flaps in dogs with corneal ulcers, affording greater comfort to patients and proving to be an excellent option for ophthalmologists.

## Introduction

Corneal ulcer or ulcerative keratitis refers to a breakdown of the corneal epithelium that exposes the underlying corneal stroma. The main clinical signs are pain, blepharospasm, epiphora, photophobia, conjunctival hyperemia, corneal edema, and ocular discharge. Diagnosis is based on clinical signs and retention of fluorescein dye on the cornea [1, 2]. Depending on the characteristics of the lesion [1, 3–5], therapeutic procedures may be clinical, surgical, or a combination of both. The conjunctival flap is the most accessible surgical procedure used to treat chronic, infected or progressive corneal ulcers, and descemetoceles [6]. Flaps were created by making two parallel incisions close to the limbus in the bulbar conjunctiva, divulging, and separating Tenon’s capsule to release the conjunctiva between the two incisions, and then sectioning one of the extremities. The conjunctival flap is then positioned and sutured to the cornea [2].

Hydrodissection is a minimally invasive procedure that involves injecting liquid into an anatomical space to facilitate dissection during surgery [7]. The use of this technique in many surgical specialties, such as thoracic surgery [8], cardiac surgery [9, 10], urology [11], orthopedics [12], general surgery [13, 14], otorhinolaryngology [15], and ophthalmology [16, 17], has been reported. Hydrodissection has been used in phacoemulsification surgery to separate the lens cortex from its capsule [18], in the periocular region to facilitate dissection and reduce bleeding [19], and in the removal of bulbar conjunctiva adhered to the cornea after the construction of a 360°C flap [20]. The procedure for creating conjunctival flaps in the eyes of normal dogs has already been evaluated [21]; however, it has not yet been described in clinical situations.

Hydrodissection is a surgical technique that aims to facilitate dissection and allows safe and less traumatic separation of anatomical planes. It has already been described the use of this technique in the construction of conjunctival flap in healthy dogs. Therefore, it is expected to ensure a higher success rate in the creation of conjunctival flaps while reducing the risk of complications. This assumption applies in particular to the divulsion of the conjunctiva, reduction of surgical time, ease of handling, control of bleeding, and better healing, which contributes to the comfort of patients with corneal ulcers and avoids complications such as suture dehiscence.

This study aimed to evaluate the use of hydrodissection in the creation of conjunctival flaps in dogs with corneal ulcers.

## Materials and Methods

### Ethical approval

This study was approved by the Ethics Committee for Animal Experimentation of the State University of Maranho (UEMA) on January 18, 2021, under Protocol No. 12/2020. The owners of the canine patients to be admitted to the study signed an informed consent form based on the guidelines of the National Health Council, set forth in Resolution CNS No. 466/2012 of the Ministry of Health.

### Study period and location

The study was conducted from March 2020 to December 2022 at the University Veterinary Hospital of the State UEMA, in the city of So Lus, state of Maranho, Brazil.

### Patients

This study focused on 17 eyes from male and 17 eyes from female dogs, aged 1–6 years, with a diagnosis of unilateral deep corneal ulcer, treated at the Veterinary Ophthalmology Service of the University Veterinary Hospital of UEMA. Ophthalmic examination was based on reflex test, Schirmer tear test, slit-lamp biomicroscopy, indirect binocular and direct monocular ophthalmoscopy, applanation tonometry, and sodium fluorescein staining. A fundoscopic examination was also carried out to exclude vitreous and retinal diseases. Patients with a clinical disease who had been recommended for conjunctival pedicle flap surgery were included in the study. The patients were divided into two groups after the evaluation. To assess systemic conditions, physical examination, complete blood count, study of liver and kidney functions, blood glucose, and electrocardiogram were performed.

### Groups

The eyes were divided into two groups, G1 and G2, and all of them underwent conjunctival flap surgery. In G1 (n = 9), the conjunctival flap was created using the hydrodissection technique, while in G2 (n = 8), the flap was constructed by conventional divulsion using scissors. All the procedures were performed by the same surgeon.

### Procedures

#### Conjunctival pedicle flap

Patients were placed in the dorsal position under routine anesthesia. A commercial aqueous solution of iodopyrrolidine was used as an antisepsis agent on the eyelids, and the same solution diluted in lactated Ringer’s solution in a 1:50 ratio was used on the ocular surface. Anesthetic eye drops based on 1% tetracaine hydrochloride and 0.1% phenylephrine hydrochloride were instilled after antisepsis, the surgical field was arranged, and mechanical blepharostasis was performed. Procedures were performed under magnification and illumination of a surgical microscope equipped with microfocus, with the ocular surface positioned parallel to the lens. G2 underwent a traditional technique involving conjunctival incision using iris scissors and blunt dissection to form the pedicle. G2 underwent hydrodissection by subconjunctival injection of 1 mL of 9% sodium chloride using a 26 G needle and a 1 mL syringe followed by dissection using scissors to create the pedicle. At the end of the procedure, the conjunctival flap was positioned over the corneal lesion, previously debrided and sutured with simple interrupted stitches with nylon 9-0 suture. Tobramycin-based antibiotic eye drops were instilled into the affected eye at 4 h intervals for 7 days.

### Assessment protocols

#### Intraoperative assessment

The operative time in each eye was recorded using a stopwatch, and the count started from the hydrodissection in G1 and from the incision in G2 until the suture of the corneal flap. The degree of bleeding was assessed by counting the number of sterile swabs that were used to tamponade the surgical field during the procedure. We also assessed the ease of handling the conjunctiva, considering the degree of difficulty in divulsion of the conjunctiva and flap creation.

### Clinical evaluations

Clinical evaluations were conducted before the surgical procedure (T0) and after 24 h on post-operative day 1 (T1), day 7 (T7), and day 14 (T14). Blepharospasm, hyperemia, and conjunctival edema [18] were examined and classified as absent, mild, moderate, or severe. A blind assessment of the conjunctival scarring was performed by a surgeon and monitored based on post-operative photographs and on a visual analog scale from 1 to 3 (1 = excellent, 2 = good, and 3 = fair). Parameters under evaluation included restitution of the conjunctival epithelium to the region of the limbus and formation or non-formation of the granulation tissue. Post-operative complications, particularly suture dehiscence, were recorded in each group at each time point.

### Statistical analysis

Parametric data were tabulated and arranged in a 2 × 4 factorial design and tested for assumptions of normality and homoscedasticity of errors. The data were subjected to an analysis of variance, and the averages were analyzed using Tukey’s test. The Wilcoxon test (between treatments) and the Kruskal–Wallis test were used to compare qualitative variables over time. p-value of 95% (p = 0.05) was considered statistically significant in all tests.

## Results

In this study, 17 eyes from 17 dogs suffering from unilateral deep focal corneal ulcers who underwent conjunctival pedicle flap surgery were treated. Table-1 describes the patient profile. As shown in Table-1, 94% of the patients were Shih Tzu dogs; 58.8% were up to 3 years old and 41.2% were up to 6 years old, 58.8% were male, and 41.2% were female.

**Table-1:** Profile of the patients in this study.

Group	Species	Breed	Sex	Age	Affected eye
G2 – without hydrodissection	Canine	Shih Tzu	F	1 year	Right
G2	Canine	Shih Tzu	M	3 years	Right
G2	Canine	Shih Tzu	M	4 years	Left
G2	Canine	Shih Tzu	F	1.3 years	Right
G2	Canine	Shih Tzu	M	1 year	Left
G2	Canine	Shih Tzu	M	1 year	Left
G2	Canine	Shih Tzu	F	2.8 years	Right
G2	Canine	Shih Tzu	F	5 years	Left
G1 – with hydrodissection	Canine	Shih Tzu	M	6 months	Right
G1	Canine	Shih Tzu	M	4 years	Left
G1	Canine	Shih Tzu	M	8 years	Right
G1	Canine	Spitz	F	6 years	Left
G1	Canine	Shih Tzu	M	5 months	Left
G1	Canine	Shih Tzu	F	6 years	Right
G1	Canine	Shih Tzu	M	6 years	Left
G1	Canine	Shih Tzu	M	3 years	Left
G1	Canine	Shih Tzu	F	2.6 years	Left

### Intraoperative assessment

**Figure-1:**
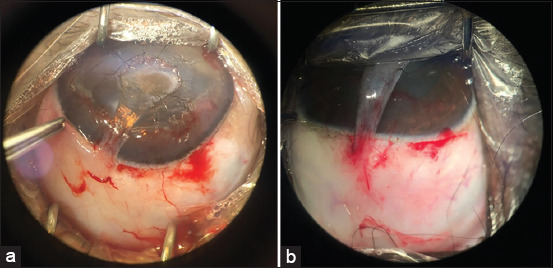
Slight bleeding observed in various patients in clinical phase II. (a) With hydrodissection. (b) Without hydrodissection. Source: Veterinary Ophthalmology Service of HVU-UEMA. 2021.

**Figure-2:**
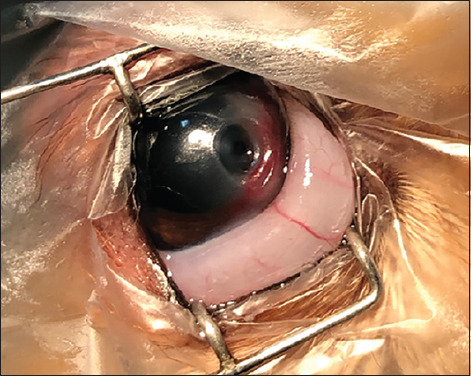
Eye affected by deep corneal ulcer after performing hydrodissection. Note the distention of the conjunctiva caused by the procedure. Source: Veterinary Ophthalmology Service of the University Veterinary Hospital – HVU-UEMA. 2021.

The mean total surgical time was 44 ± 10.5 min in G1 and 13.4 min in G2. There was no statistical difference between the two groups (Table-2). With regard to bleeding, in the group with hydrodissection a swab was used for hemostasis in nine eyes (100%), with a median of 1 swab. In the group without hydrodissection, a swab was used for hemostasis in five eyes (62.5%), with a median of 0.5 swabs (Figure-1). No statistical difference was found between the two groups (Table-3). Handling the conjunctiva was considered easy in 8/9 (88.8%) and 7/8 (87.5%) patients in the G1 and G2 groups. There were no differences in this parameter between the groups (Table-4). The volume of 0.9% sodium chloride administered to the conjunctiva ranged from 0.5 mL to 1 mL, with a mean value of 0.74 mL and a median value of 0.7 mL in G1 (Figure-2).

**Table-2:** Mean, standard deviation, and coefficient of variation of surgical time spent on affected eyes.

Group	Variable	Mean	Standard deviation	Coefficient of variation (%)
Without hydrodissection	Surgical time	44^A^	13.4	35.1
With hydrodissection		44^A^	10.5	30.4

Means followed by the same letters do not differ from each other by Tukey’s test at p > 0.05. Data with normal distribution of errors by the Cramer-Von Mises test (W = 0.07; p > 0.25)

**Table-3:** Median number of swabs used for hemostasis on the affected eyes.

Group	Variable	Median
Without hydrodissection	Bleeding	0.5^A^
With hydrodissection		1^A^

Medians followed by the same letter do not differ from each other according to Wilcoxon’s test at p = 0.39

**Table-4:** Median ease of handling of the conjunctiva of affected eyes.

Group	Variable	Median
Without hydrodissection	Ease of handling	1
With hydrodissection		1

There were no differences between treatments according to the Wilcoxon test at p > 0.05

### Post-operative evaluation

**Figure-3:**
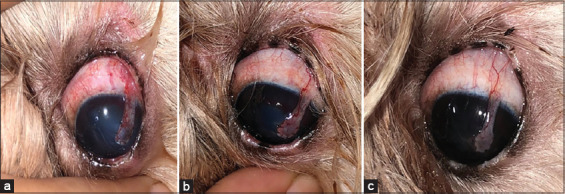
Canine patient. Left eye. Wound healing of the conjunctiva of a patient in G1 (with hydrodissection). Phase II. (a) Post-operative day 1; (b) Post-operative day 7; and (c) Post-operative day 14. Source: Veterinary Ophthalmology Service of HVU-UEMA. 2021.

**Figure-4:**
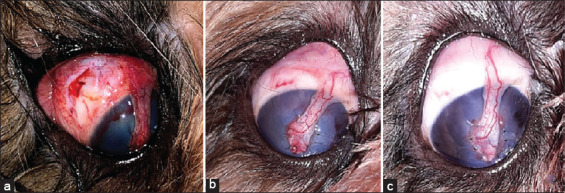
Canine patient. Right eye. Wound healing of the conjunctiva of a patient in G2 (without hydrodissection). Phase II. (a) Post-operative day 1; (b) Post-operative day 7; and (c) Post-operative day 14. Source: Veterinary Ophthalmology Service of HVU-UEMA. 2021.

Blepharospasm was observed preoperatively and 24 h after the procedure, which disappeared by post-operative day 7. Hyperemia was observed preoperatively to 7 days postoperatively, but it cleared up by day 14 in both groups. Conjunctival edema, which had disappeared by post-operative day 14 (Table-5), was observed in G1 and G2. The patients in this study showed satisfactory healing of the conjunctival flap donor site. Scarring gradually improved over the first 7 post-operative days, and by post-operative day 14, all the patients exhibited excellent healing according to the visual analog scale (Figures-3 and 4). There was no statistically significant difference between the two groups (Table-6). Dehiscence of flap sutures occurred in 4/17 (23.5%), 2/9 (22.2%), and 2/8 (25%) patients in the G1 and G2 groups, respectively, with no statistically significant difference between the groups.

**Table-5:** Values of blepharospasm, conjunctival hyperemia, and edema in the groups and surgical times spent on the affected eyes.

Group	Variable	Times (days)				p-value

		0	1	7	14	
Without hydrodissection	Blepharospasm	1	1*	0*	0*	0.002
With hydrodissection		1	1*	0*	0*	0.001
Without hydrodissection	Hyperemia	1	1	1	0	0.06
With hydrodissection		1	1.5	1	0*	0.002
Without hydrodissection	Conjunctival Edema	0	0	0.5	0	0.08
With hydrodissection		0	1*	0	0	0.002

* Medians differing from the initial time (time 0), according to the Kruskal–Wallis test. There were no differences between treatments, according to the Wilcoxon test at p > 0.05. 0-Immediate postop period; 1-Post-operative day 1; 7-Post-operative day 7; 14-Post-operative day 14.

**Table-6:** Wound healing of the conjunctiva of the two groups and times evaluated (in days).

Group	Variable	Times (days)			p-value

		1	7	14	
Without hydrodissection	Wound healing	2.5	2	1.5*	0.01
With hydrodissection		2	2	1*	0.001

* Medians differing from the initial time (time 0), according to the Kruskal–Wallis test. There were no differences between treatments, according to the Wilcoxon test at p > 0.05. 1-Post-operative day 1; 7-Post-operative day 7; 14-Post-operative day 14.

## Discussion

Numerous studies have described the use of hydrodissection in different surgical interventions in humans, which facilitates dissection, reduces surgical time, requires less manipulation, and thus improves the clinical outcomes of patients [22–25]. However, hydrodissection has rarely been described in veterinary medicine, especially in conjunctival procedures.

Hydrodissection has been described for the first time in the construction of conjunctival flaps in normal dogs, and the authors concluded that it is an easy procedure that optimizes the creation of conjunctival flaps, thus becoming a new option for surgeons, especially for beginning ophthalmologists [21]. However, we believe that it is important to continue studies of conjunctival flap construction in routine clinical situations.

As regards the patient profile, it was found that Shih Tzu dogs were mainly affected by corneal ulcers. This confirms previous reports that this dog breed is most commonly affected by ophthalmic disorders, particularly corneal ulcers [1, 26].

There was no difference in the operative time between the groups, demonstrating that both procedures are effective, and the surgical time is the same. Operative time is an important parameter that should be considered during surgical procedures. Longer operative time significantly contributes to the risk of complications, particularly infection [27]. The operative time spent in the construction of conjunctival flaps in normal dogs was analyzed, and no change was observed [21]. Several factors, such as pre-operative planning, surgeon experience, and fatigue, may prolong the operative duration [28]. However, it should be noted that the procedures were performed by the same veterinary surgeon who earned his degree from the Brazilian College of Veterinary Ophthalmology and whose learning curve in this technique has reached a plateau, thus minimizing this type of influence on the results of this research.

Transoperative bleeding was negligible, and the groups had no statistically significant difference (p = 0.39). Clinical phase I trials involving conjunctival hydrodissection also did not show statistical differences in this parameter [21]. Bleeding, a commonly evaluated parameter in hydrodissection trials, is variable. Transoperative bleeding in humans undergoing facial lipoplasty with and without hydrodissection has also been reported as not statistically different, as all the patients bled <5 mL intraoperatively, which is equivalent to 1 gauze pad [25].

Patients who underwent laparoscopic cholecystectomy using hydrodissection and conventional techniques also showed no change in the average dissection time and bleeding from the liver [29]. These findings were corroborated by the results of this study.

Conjunctival flap construction was considered “easy,” indicating that both procedures were effective in handling the conjunctiva in both groups. In the case of a few patients, the procedure was more difficult due to the presence of ocular conditions involving varying degrees of inflammation and chronic disease. Ulcers present in different ways, leading to conjunctival consequences such as increased vascularity, edema, and fibrosis [2]. In some cases, these factors made the procedure more difficult. However, in general, these procedures were performed without major limitations in both groups.

The volume of liquid used for hydrodissection varies considerably. According to the literatures, different volumes are used depending on the medical specialty [25, 30, 31]. Galeno *et al*. [21] created conjunctival flaps in normal dogs using a volume of 0.7 mL. The subconjunctival route involves the use of volumes similar to those described in this study, although for treating different conditions [32–35].

Blepharospasm occurs if the patient does not open his eyelids freely. This symptom, which is usually caused by ocular pain, may occur when there are injuries to the cornea and conjunctiva, alterations in eyelashes and eyelids, and glaucoma [2]. In the present study, mild blepharospasm was observed 24 h after the surgical procedure, which was attributed to discomfort caused by the ulcer and surgical manipulation of the cornea.

Most of the patients in this study exhibited mild conjunctival hyperemia due to trauma caused by manipulation of the conjunctiva as well as due to the condition of the ulcer itself. Hyperemia presents as an increase in arterial blood flow to the conjunctiva in response to simple or complex injuries [5]. The conjunctiva is a thin, exposed mucous membrane with high vascularity and lymphoid content that readily responds to harmful stimuli [36].

Conjunctival edema or chemosis refers to swelling of the conjunctiva in response to accidents or surgical trauma [2]. This parameter presented mildly 24 h after the surgical procedure in several patients in both G1 and G2, and its presence was the result of surgical trauma of the conjunctiva.

The healing aspect of the conjunctiva was considered excellent in the patients of this study, indicating that despite the injury generated in the conjunctiva by the construction of the flap, healing was satisfactory and without complications. Conjunctival tissue has a high capacity for repairing conjunctival injuries, enabling them to heal naturally [37], a fact that was confirmed in this study. Galeno *et al*. [21] also reported satisfactory healing of conjunctival wounds in normal dogs subjected to the construction of conjunctival flaps, corroborating the findings of this clinical study.

## Conclusion

Conjunctival hydrodissection is an easy technique that can facilitate the construction of conjunctival flaps in dogs with corneal ulcers, thereby ensuring greater comfort to patients, and facilitating the task of the ophthalmologist.

## Authors’ Contributions

LSG: Conducted the study and wrote the manuscript with input from all authors. ARSL: Data collection and evaluation of patients. JRSJ: Analyzed the data. ALA and TBL: Conceived, supervised, designed and implemented the study. All authors have read, reviewed, and approved the final manuscript.
